# Supplementary material for: Altered grid-like coding in early blind people
Source: Nat Commun. 2024 Apr 24;15:3476. doi: 10.1038/s41467-024-47747-x (PMC11043432; doi:10.1038/s41467-024-47747-x)
Supplement: Supplementary file 1 — Supplementary Information [file 41467_2024_47747_MOESM1_ESM.pdf]

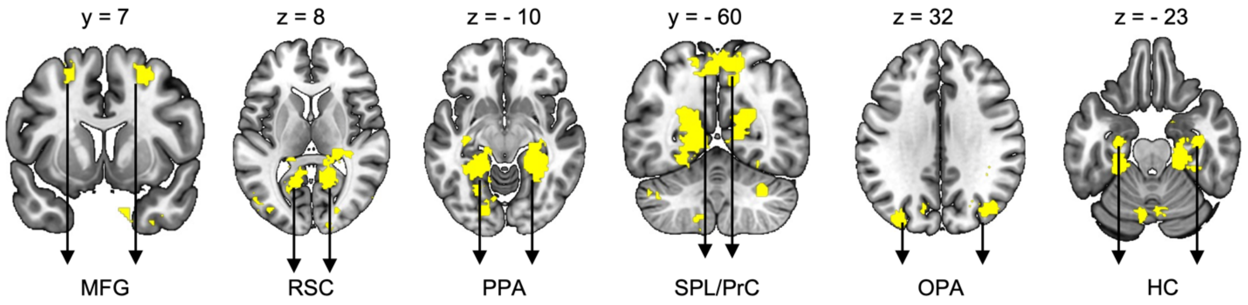

Figure S1. The brain mask of the human navigation network

The mask of the human navigation network consisted of (bilateral) medial frontal gyrus (MFG), retrosplenial cortex (RSC), parahippocampal place area (PPA), superior parietal lobe/precuneus (SPL/PrC), occipital place area (OPA), and hippocampus (HC). The mask was obtained from the term-based meta-analysis on *Neurosynth*[1] across 77 studies ($p_{fdr} < 0.01$, the term was "navigation"). The mask is overlapped with the MNI-152 T1 template.

Supplementary Table 1. Brain regions more active in the navigation task than in the math task ($p_{FDR} < 0.05$)

Brain Regions	MNI Coordinates of the Peak Voxel			Peak T Value
	X	Y	Z	
**Sighted Controls**				
Left Middle Frontal Gyrus	-21	8	53	4.5
Right Middle Frontal Gyrus	24	2	53	5.89
Left Retrosplenial Cortex	-9	-46	8	5.29
Right Retrosplenial Cortex	15	-55	11	5.85
Left Parahippocampal Place Area	-27	-43	-10	4.14
Right Parahippocampal Place Area	27	-37	-13	4.73
Left Superior Parietal Lobe/Precuneus	-12	-70	53	7.04
Left Occipital Place Area	-33	-82	32	5.47
Right Occipital Place Area	36	-76	17	5.54
**Early Blind Individuals**				
Left Middle Frontal Gyrus	-21	4	56	3.03
Right Middle Frontal Gyrus	24	5	50	3.70
Left Retrosplenial Cortex	-12	-55	11	3.73
Right Retrosplenial Cortex	21	-52	5	4.62
Left Parahippocampal Place Area	-33	-37	-16	3.29
Right Parahippocampal Place Area	30	-43	-10	3.09
Left Superior Parietal Lobe/Precuneus	-15	-70	56	4.89
Left Occipital Place Area	-33	-85	20	4.04
Right Occipital Place Area	42	-79	11	4.10

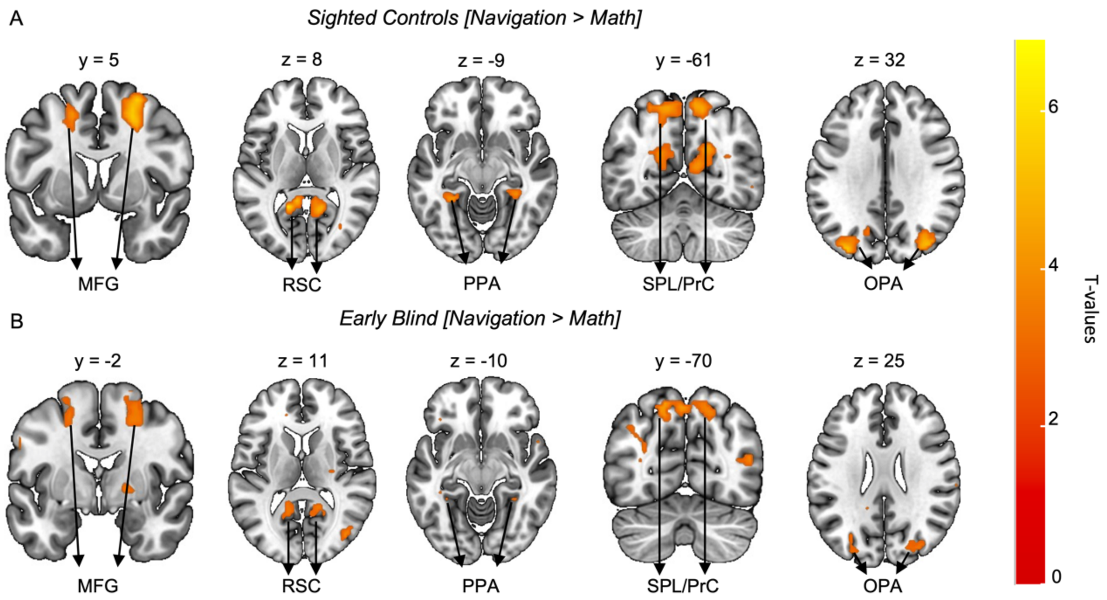

Figure S2. Results of Navigation vs. Math contrast without the predefined mask

Whole-brain results of the Nav-Math experiment demonstrated that both sighted controls (A, n = 19) and early blind individuals (B, n = 19) activated the same network of regions (i.e., the human navigation network) during the imagined navigation of the clock space (Navigation > Math). The activations were thresholded at $p < 0.01$ uncorrected and overlapped on the MNI-152 T1 template.

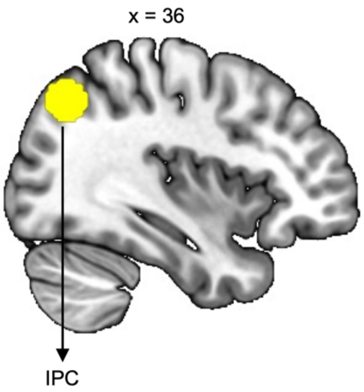

Figure S3. Early blind individuals relied more on the inferior parietal cortex (IPC) during navigation than SC.

Small Volume correction analyses were performed with a 10mm sphere around the peak coordinates from an independent study (MNI coordinates: 36/-68/44, Schindler et al., 2013), which investigated egocentric representation during an imagined navigation task. Results demonstrate a significant activation within the region of interest, suggesting that the early blind individuals (n = 19), compared to the sighted controls (n = 19), had greater activation in the IPC during the imagined navigation task than the math task ($p_{fwe} < 0.05$; i.e., [Navigation > Math] × [early blind > sighted controls]).

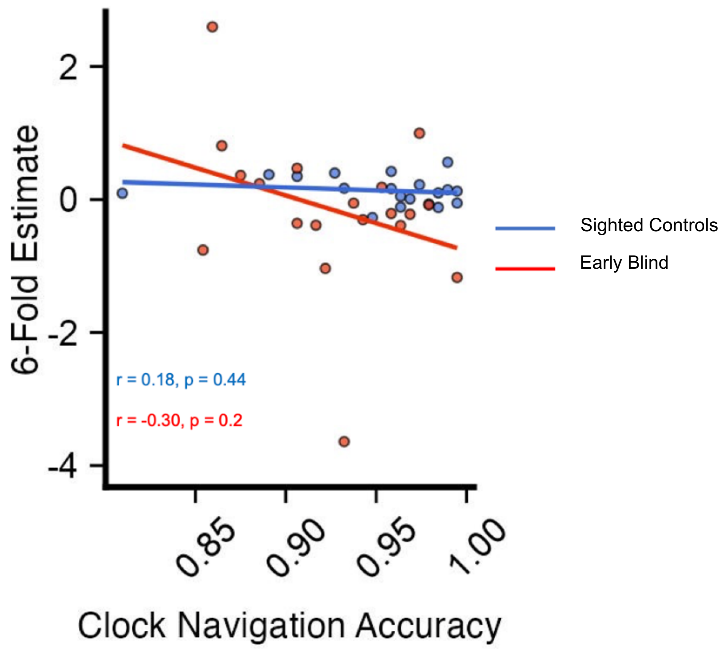

Figure S4. Correlation between the accuracy in the Clock Navigation Experiment and 6-fold grid-like coding

We did not find significant evidence that the accuracy in the Clock Navigation experiment correlated with the 6-fold symmetry estimate (Left EC for sighted controls and bilateral EC for early blind individuals), neither in sighted controls ($n = 19$, $r = -0.18$, $p = 0.44$, $r^2 = 0.03$) nor in early blind individuals ($n = 19$, $r = -0.30$, $p = 0.2$, $r^2 = 0.09$). However, it is worth noting that, although not significant, there was a negative trend in the early blind group, where participants with the higher accuracy scores also expressed a lower grid-like coding in the EC. This result support the hypothesis that the reduced 6-fold signal in the early blind was not attributable to the difference in task performance between the two groups. Source data are provided as a Source Data file.

Supplementary Table 2. <u>4-fold Symmetry Score Predicted by IPC Activity</u>

Linear regression

**Coefficients**	**Estimate**	**Std. Error**	**T-value**	**Pr (>ltl)**
Intercept	0.311	0.085	3.656	0.0008***
IPC Activity	0.153	0.052	2.95	0.005**
Group	- 0.41	0.12	- 3.4	0.0017**
IPC Activity : Group	- 0.18	0.07	- 2.33	0.02*

$R^2 = 0.38$; Adjusted $R^2 = 0.32$; $F(3,34) = 6.95$; $p = 0.0009$. All p-values are two-tailed

Source data are provided as a Source Data file.

Supplementary Table 3. Path information in the path integration task

START	FIRST STOP	SECOND STOP	DISTANCE: START-FIRST	DISTANCE: START-SECOND	ANGLE (θ_1) START-FIRST	ANGLE (θ_2) START-SECOND
N	D	O	14.5	4.1	34.7°	107.8°
I	C	A	7.2	10.15	53.7°	31.7°
G	O	Q	7.6	11.5	50.5°	33.7
G	P	C	10.4	7.2	37.9°	115.5°
I	Q	O	6.5	7.6	77.9°	57.3°
B	P	H	14.4	7.2	55.3°	55.3°
O	D	L	12.5	8.4	19.1°	40.6°
N	I	O	10.4	4.1	73.7°	122.7°

θ is the inner angle of the performed segment (starting point – stopping point).

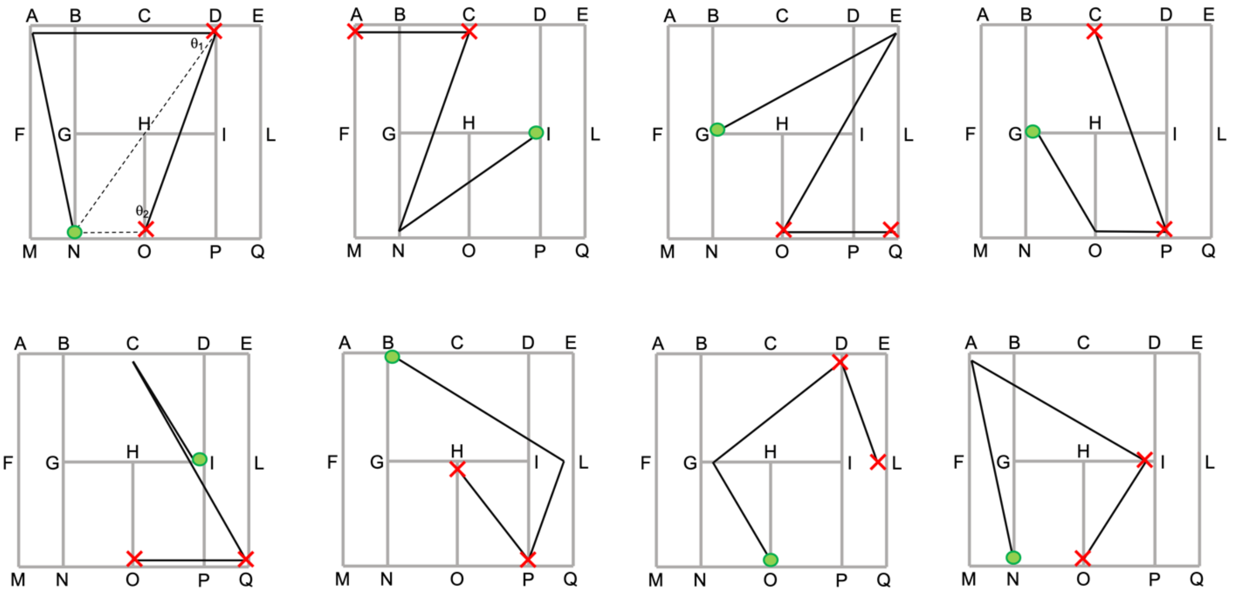

Figure S5. Illustrations of the paths in the path integration task.
Participants performed eight unique paths, repeated twice throughout the experiment. Each path was constituted by a starting point (green dots) and two different stopping points (red crosses). At each stopping point, blindfolded participants were required to estimate the distance and orientation of the starting point compared to their own position. The details of the paths are described in Supplementary Table 3.

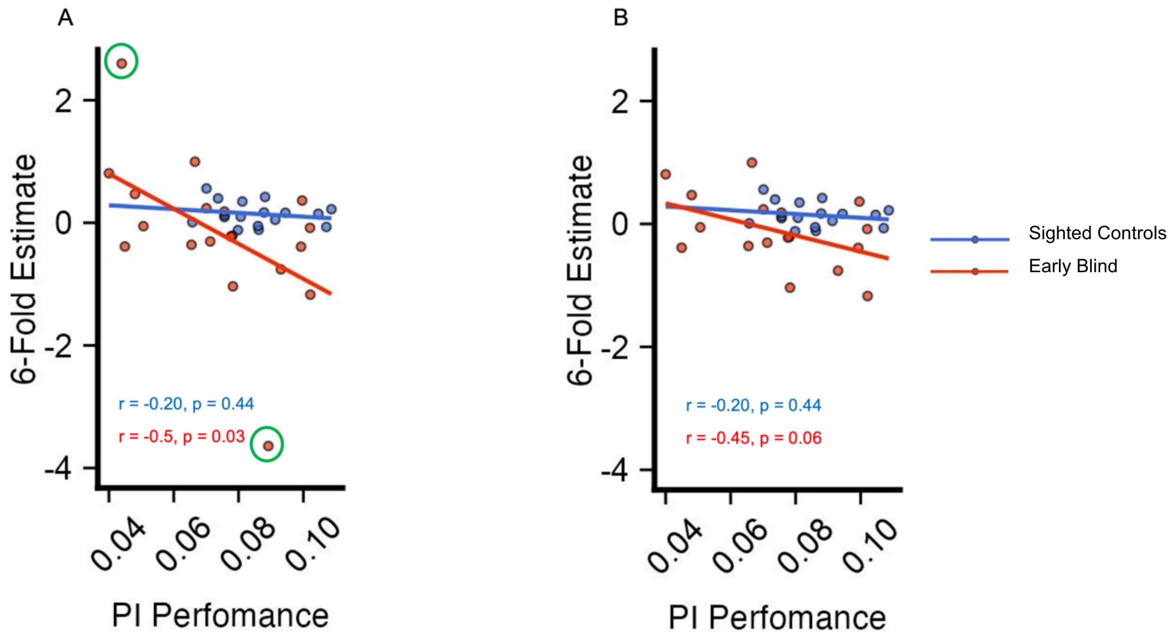

Figure S6. Path Integration (PI) ability negatively correlated with 6-fold symmetry in Early Blind participants.

(A) 6-Fold symmetry estimates (Left EC for sighted controls and bilateral EC for early blind individuals) negatively correlated with PI Performance in early blind participants, suggesting that sighted-like representation of space might be dysfunctional for navigating without vision (Pearson's product-moment correlations; n = 19, r = -0.5, p = 0.03). However, this correlation might be biased by the presence of two outliers (green circles) in the early blind population. No significant correlation was found in the sighted control group (n = 17, r = -0.19, p = 0.44). (B) Removing the outliers in the early blind group weakened the negative correlation between 6-fold symmetry estimates and PI performance (n = 17, r = -0.45, p = 0.06). Source data are provided as a Source Data file.

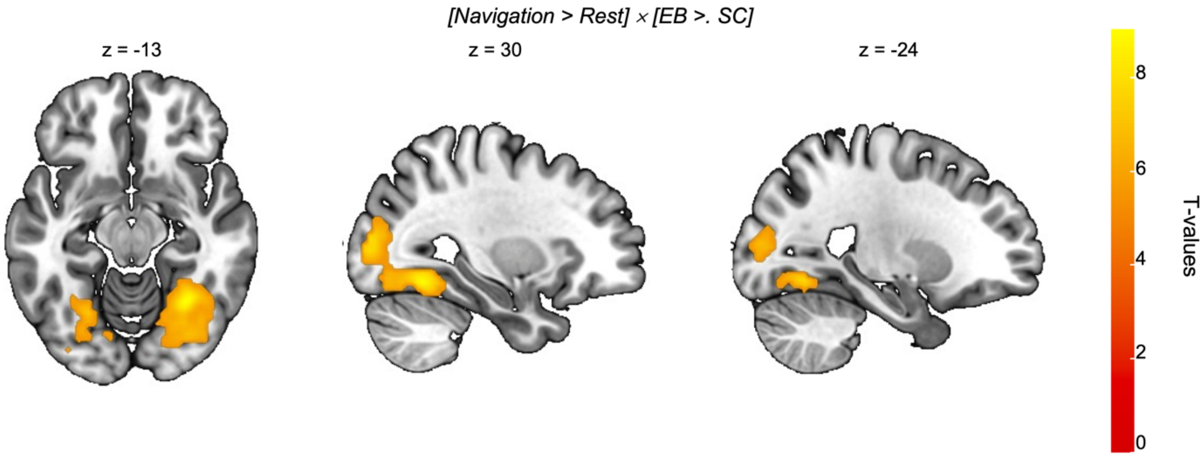

Figure S7. The occipital cortex was more activated in early blind than in sighted controls. Although no differences between sighted controls (n = 19) and early blind individuals (n = 19) were detected when the navigation task was compared to the math task, when comparing the navigation task against rest ([Navigation > Rest] × [early blind > sighted controls]), we could find the emergence of clusters of activity in several occipital areas among which bilateral inferior occipital gyrus; occipital fusiform gyrus; fusiform gyrus and lingual gyrus in early blind individuals more than sighted controls, which is in line with previous results[2,3] (The activations were thresholded at $p_{FWE} < 0.05$ and overlapped on the MNI-152 T1 template).

Supplementary Table 4. Strategies for imagined navigation during the Clock Navigation experiment.

Code	Strategies
**Sighted Controls (SC)**	
SC01	Thinking about the path I need to perform
SC02	Divide the clock in two halves by tracing a line from the first to the second point
SC03	Walk through the clock from the first to the second number
SC04	Bird-view of the clock to locate the starting and ending position
SC05	Walk through the clock from the starting to the ending point
SC06	Divide the clock space with a line
SC07	Visualize the clock from the starting point
SC08	Walk through the clock
SC09	Rotate the clock space according to my position
SC10	Rotate the clock space according to the starting point position
SC11	Bird-view of the clock
SC12	Divide the clock in two halves from the starting to the ending point
SC13	Walk through the clock
SC14	Bird-view of the clock space tracing a line from the starting to the ending point
SC15	Imagine the path to perform within the clock
SC16	Walk through the clock
SC17	Imagine walking from one number to the other
SC18	Walk through the clock
SC19	Imagine walking from one point to the other of the space
**Early Blind (EB)**	
EB01	Trace a line from one point to the other
EB02	Rotate the clock according to the starting point location and walk until the target point
EB03	Imagine always looking at the ending point being at the starting position
EB04	Imagine having the ending point in front of me
EB05	Imagine two points on the clock
EB06	Rotate the clock to have the starting point in front of me
EB07	Imagine myself within the clock space
EB08	Imagine the clock space divided in two halves according to the starting and ending point
EB09	Rotate the clock to have the starting point in front of me
EB10	Rotate the clock to always face the ending point
EB11	Walk from one number to the other
EB12	Imagine the path to perform
EB13	Rotate the clock to have the starting point in front of me
EB14	Rotate the clock to have the starting point in front of me
EB15	Rotate the clock to have the starting point in front of me
EB16	Imagine the ending point in front of me
EB17	Divide the clock with a line
EB18	Imagine the ending point in front of me
EB19	Imagine the ending point in front of me

Supplementary Table 5. Demographic information of the early blind individuals (EB) and their matched sighted controls (SC)

EB CODE	AGE RANGE	GENDER	ONSET OF TOTAL BLINDNESS	ETIOLOGY	SC CODE	AGE RANGE	GENDER
EB01	46-56	M	Birth	Optic nerve hypoplasia	SC01	39-49	M
EB02	31-41	M	Birth	Retinitis pigmentosa	SC02	27-37	M
EB03	39-49	M	Birth	Retinal burnt in the incubator	SC03	38-48	M
EB04	28-38	F	Birth	Microphthalmia	SC04	24-34	F
EB05	31-41	F	Birth	Premature retinopathy	SC05	28-38	F
EB06	29-39	F	Birth	Bilateral aplasia	SC06	26-36	F
EB07	37-47	F	Birth	Retrolenticular fibroplasia	SC07	40-50	F
EB08	29-39	M	Birth	Leber congenital amaurosis	SC08	26-36	M
EB09	29-39	F	8 months	Congenital retinitis pigmentosa	SC09	31-41	F
EB10	32-42	M	Birth	Bilateral congenital anophthalmos	SC10	36-46	M
EB11	30-40	F	2 years	Bilateral retinoblastoma	SC11	26-36	F
EB12	27-37	F	Birth	Premature retinopathy	SC12	25-35	F
EB13	29-39	M	Birth	Premature retinopathy	SC13	30-40	M
EB14	33-43	M	Birth	Optic nerve hypoplasia	SC14	29-39	M
EB15	30-40	F	Birth	Congenital retinitis pigmentosa	SC15	32-42	F
EB16	32-42	F	Birth	Premature retinopathy	SC16	29-39	F
EB17	24-34	M	5 years	Dominant optic atrophy	SC17	25-30	M
EB18	31-41	F	Birth	Premature retinopathy	SC18	34-44	F
EB19	48-58	M	Birth	Bilateral congenital glaucoma	SC19	48-58	M

Supplementary References

1. Yarkoni, T., Poldrack, R. A., Nichols, T. E., Van Essen, D. C. & Wager, T. D. Large-scale automated synthesis of human functional neuroimaging data. *Nat. Methods* **8**, 665–670 (2011).

2. Gagnon, L. *et al*. Activation of the hippocampal complex during tactile maze solving in congenitally blind subjects. *Neuropsychologia* **50**, 1663–1671 (2012).

3. Kupers, R., Chebat, D. R., Madsen, K. H., Paulson, O. B. & Ptito, M. Neural correlates of virtual route recognition in congenital blindness. *Proc. Natl. Acad. Sci. U. S. A.* **107**, 12716–12721 (2010).
