# Supplementary material for: Altered grid-like coding in early blind people
Source: Nat Commun. 2024 Apr 24;15:3476. doi: 10.1038/s41467-024-47747-x (PMC11043432; doi:10.1038/s41467-024-47747-x)
Supplement: Supplementary file 3 — Reporting Summary [file 41467_2024_47747_MOESM3_ESM.pdf]

nature portfolio

Corresponding author(s): Federica Sigismondi
Roberto Bottini

Last updated by author(s): Feb 26, 2024

Reporting Summary

Nature Portfolio wishes to improve the reproducibility of the work that we publish. This form provides structure for consistency and transparency in reporting. For further information on Nature Portfolio policies, see our Editorial Policies and the Editorial Policy Checklist.

Statistics

For all statistical analyses, confirm that the following items are present in the figure legend, table legend, main text, or Methods section.

n/a	Confirmed	
☐	☒	The exact sample size (n) for each experimental group/condition, given as a discrete number and unit of measurement
☐	☒	A statement on whether measurements were taken from distinct samples or whether the same sample was measured repeatedly
☐	☒	The statistical test(s) used AND whether they are one- or two-sided<br>*Only common tests should be described solely by name; describe more complex techniques in the Methods section.*
☐	☒	A description of all covariates tested
☐	☒	A description of any assumptions or corrections, such as tests of normality and adjustment for multiple comparisons
☐	☒	A full description of the statistical parameters including central tendency (e.g. means) or other basic estimates (e.g. regression coefficient) AND variation (e.g. standard deviation) or associated estimates of uncertainty (e.g. confidence intervals)
☐	☒	For null hypothesis testing, the test statistic (e.g. F, t, r) with confidence intervals, effect sizes, degrees of freedom and P value noted<br>*Give P values as exact values whenever suitable.*
☒	☐	For Bayesian analysis, information on the choice of priors and Markov chain Monte Carlo settings
☒	☐	For hierarchical and complex designs, identification of the appropriate level for tests and full reporting of outcomes
☐	☒	Estimates of effect sizes (e.g. Cohen's d, Pearson's r), indicating how they were calculated

Our web collection on statistics for biologists contains articles on many of the points above.

Software and code

Policy information about availability of computer code

Data collection	The data were collected at the Center for Mind/Brain Sciences (CIMeC, University of Trento) using a 3T Siemens Prisma Scanner equipped with a 64-channel headcoil. The experiments (fMRI and Behavioral) were implemented using Matlab v2017 and Psychtoolbox 3.0.14.
Data analysis	Different software were used to analyze the data: SPM (v12, https://www.fil.ion.ucl.ac.uk/spm/software/spm12/) to compute the univariate analyses of the Nav-Math experiment and the hexadirectional coding analyses of the Clock-Navigation experiment; R v4.2.2 to compute behavioral and Region of Interest analyses; and Freesurfer (v7.1.1, https://surfer.nmr.mgh.harvard.edu/) to create subject-specific entorhinal cortex ROI. Custom codes are available online at https://doi.org/10.5281/zenodo.10694439

For manuscripts utilizing custom algorithms or software that are central to the research but not yet described in published literature, software must be made available to editors and reviewers. We strongly encourage code deposition in a community repository (e.g. GitHub). See the Nature Portfolio guidelines for submitting code & software for further information.

Data

Policy information about availability of data

All manuscripts must include a data availability statement. This statement should provide the following information, where applicable:

- Accession codes, unique identifiers, or web links for publicly available datasets
- A description of any restrictions on data availability
- For clinical datasets or third party data, please ensure that the statement adheres to our policy

The neuroimaging and behavioral data generated in this study have been deposited in the Zenodo database under accession code https://doi.org/10.5281/

zenodo.1069443980. The raw and pre-processed neuroimaging data are protected and are not available due to data privacy laws, access can be obtained by forwarding a formal inquiry to the corresponding author (federica.sigismondi@unitn.it).

Research involving human participants, their data, or biological material

Policy information about studies with human participants or human data. See also policy information about sex, gender (identity/presentation), and sexual orientation and race, ethnicity and racism.

Reporting on sex and gender	Thirty-eight participants of both sexes took part in the fMRI experiment (19 Sighted controls, 10 females; 19 Early Blind, 10 females). Thirty-six participants took part at the behavioral Path Integration experiment (17 Sighted controls, 8 females and 19 Early Blind, 10 females). We did not perform any sex-based analyses and thus, we suggest that the findings are applicable to both sexes. Sex was determined by self-report.
Reporting on race, ethnicity, or other socially relevant groupings	n/a
Population characteristics	Early blind participants (M = 37.37, SD = 6.13) were overall matched by sex and age with Sighted Controls participants (fMRI experiments: M = 36.21, SD = 6.44, Behavioral Path Integration: M = 35.88, SD = 6.41). Blind individuals became blind at birth or within the 5th years of life, reporting, at most, faint light perception and no visual memory. All participants reported not to have any neurological or psychiatric disorder and all fluently speak Italian. Participants signed an informed consent and were compensated for their participation.
Recruitment	Sighted Controls individuals were recruited from dedicated social networks groups of the University of Trento. Early blind individuals were recruited through blind organizations all over Italy. There were no self-selection biases, as the only recruitment criteria was the participants' eligibility to participate to an MRI experiment. Moreover, given the scope of the study, early blind individuals were required not to have any residual vision (e.g., shape perception) or visual memory.
Ethics oversight	This study was approved by the ethical committee of the University of Trento.

Note that full information on the approval of the study protocol must also be provided in the manuscript.

Field-specific reporting

Please select the one below that is the best fit for your research. If you are not sure, read the appropriate sections before making your selection.

☒ Life sciences ☐ Behavioural & social sciences ☐ Ecological, evolutionary & environmental sciences

For a reference copy of the document with all sections, see nature.com/documents/nr-reporting-summary-flat.pdf

Life sciences study design

All studies must disclose on these points even when the disclosure is negative.

Sample size	No sample-size calculation was performed given the peculiarity of the population tested (i.e., Early Blind) and the difficulty of accessing it. We, thus, collected as much data as possible, given early blind individuals availability.
Data exclusions	No data were excluded from the analyses
Replication	Our study replicate two previous findings (Horner et. al., 2016; Bellmund et al., 2016) on the emergence of a grid-like coding in sighted individuals' entorhinal cortex during Imagined spatial Navigation tasks. All attemps to replicate these results were succesfull
Randomization	randomization was not applicable as this is an observational study. The group factor (i.e., whether the participants were early blind or sighted individuals) is determined by nature and can not be randomized
Blinding	Blinding was not possible as the group variable (whether the participants were sighted or early blind individuals) was obvious for both the experimenter and the participants.

Reporting for specific materials, systems and methods

We require information from authors about some types of materials, experimental systems and methods used in many studies. Here, indicate whether each material, system or method listed is relevant to your study. If you are not sure if a list item applies to your research, read the appropriate section before selecting a response.

Materials & experimental systems

n/a	Involved in the study
☒	☐ Antibodies
☒	☐ Eukaryotic cell lines
☒	☐ Palaeontology and archaeology
☒	☐ Animals and other organisms
☒	☐ Clinical data
☒	☐ Dual use research of concern
☒	☐ Plants

Methods

n/a	Involved in the study
☒	☐ ChIP-seq
☒	☐ Flow cytometry
☐	☒ MRI-based neuroimaging

Magnetic resonance imaging

Experimental design

Design type

Nav-Math experiment: Block design; Clock Navigation Experiment: Event-related design

Design specifications

Each participants underwent one fMRI session. During the session participants performed two runs of the Nav-Math experiment and eight runs of the Clock navigation experiment, designed as follow:

Nav-Math Experiment: in each run, participants performed two blocks of the a spatial navigation task and two blocks of an arithmetic task. During the spatial navigation task, they were asked to imagine navigating within a clock-like environment, moving from a number to the other following the auditory instructions. Participants heard three numbers (i.e. the starting point, the ending point, and the target number, 0.5 secs each, with an inter-stimulus interval of 0.25 secs) and were asked to decided whether the target number was situated on their left or right within a time-window of 3secs. Similarly, during the math task, participants heard a target number at the beginning of each block (0.5 secs with an inter-stimulus interval of 2 secs) followed by three other numbers (0.5 secs each, with an inter-stimulus interval of 0.25 secs). They were required to sum the first two numbers, subtract the third number from the results and decide whether the final result of the operation was greater or smaller compared to the target number,within a time window of 3 secs.

Clock Navigation Experiment: in each run, participants were required to imagine to move within a clock-like environment (see above). Participants heard two numbers (i.e. the starting and the ending point, 0.5secs each) and after a jittered imagination period (4-6 secs) a target number (0.5 secs). Participants were asked to decide whether the target number was situated on the left or on the right side of the space. The time window for responses was set between 4.5 and 6.5 secs, however, participants were trained to give responses before a sound cue was played (2 secs after the target number was played).

Behavioral performance measures

Nav-Math and Clock-Navigation experiment: behavioral performance was quantified as the number of correct responses to the control question (i.e., left or right position of the target number within the clock space).

Acquisition

Imaging type(s)

functional and structural images

Field strength

3 Tesla

Sequence & imaging parameters

Echo-Planar images (EPI) was collected at the Center for Mind/Brain Sciences (CIMeC) using a Siemens Prisma scanner equipped with a 64-channel headcoil using the following parameters: Slice tilting: 15˚ from ACPC; Field of View (FoV) = 200mm; Voxel Size = 3x3x3mm; Number of slices: 66; Time Repetition (TR) = 1000ms; Time Echo (TE) = 28ms; Multiband acceleration (MB) factor = 6 and a flip angle of 59°. In addition, we've collected gradient-echo Field Maps used for distortion correction with the following parameters: FoV = 200mm; Voxel Size = 3x3x3mm3; TR: 768ms; TE = 4.92 and flip angle of 60°. Laslty we've collected T1-weighted images relying on a Multi-Echo MPRAGE: FoV = 256mm; Voxel Size = 1x1x1; mm; TR = 2530 ms; TE1 = 1.69ms; TE2 = 3.55ms; TE3 = 5.41ms; TE4 = 7.27ms and a flip angle of 7°.

Area of acquisition

whole-brain

Diffusion MRI ☐ Used ☒ Not used

Preprocessing

Preprocessing software

fMRI data were preprocessed using Statistical Parametric Mapping softwar (SPM, https://www.fil.ion.ucl.ac.uk/spm/software/spm12/) together with Matlab v2020a. Functional images were spatially realigned to first image of the first run. For each participant a voxel-displacement matrix was computed using the acquired field maps to correct for distortions. Strcutural images were realigned to the mean of functional images. Finally, functional images were normalized, and smoothed with a 6 mm full-width-at-half-maximum (FWHM) spatial kernel. Whole brain analyses were computed on normalized and smoothed images. Region of interest analyses were performed on unsmoothed images in the native space.

Normalization | Functional and structural images were normalized using unified segmentation methods to the Montreal Neurological Institute (MNI) space.

Normalization template | MNI-152

Noise and artifact removal | Potential distortions were corrected by applying to functional images the voxel displacement matrix computed from the Field Maps using the 'Realign and Unwarp' tool in SPM.

Volume censoring | No volume was censored.

Statistical modeling & inference

Model type and settings

Nav-Math experiment: for each participant the first level GLM included 5 conditions, namely the navigation instructions, mathematic instructions, mathematic target number, navigation task (regressor of interest), and mathematic task (regressor of interest). Slow drift of the signal was remove by using a high-pass filter of 1/256Hz and the six-rigid head motion parameters obtained from the preprocessing were added as regressors to account for head motions. The duration of the navigation and the math task were computed from the onset of the first number until participants' responses. At the group-level, non parametric permutation analysis was performed on the following contrasts: Navigation > Math; Math > Navigation; Navigation > Rest and Math > Rest both within and between groups (within the EB and SC group: one-sample t-test; and between EB and SC group: sighted controls > early blind and early blind > sighted controls, two-sample t-test).

Clock Navigation experiment: for each participant a four-way crossvalidation approach was applied to the quadrature filter analyses which comprehended two GLMs: GLM1 for preferred grid orientation estimation (3 partitions including 6 Runs) and GLM2 for testing each participants grid orientation (1 partition including 2 Runs). Slow-drift in the signal were addressed applying a high-pass filter 1/128Hz and six-rigid head motion parameters was introduced in the model to address head motions. Subject-specific entorhinal cortex ROIs were used to extract beta estimation obtained from the GLM2. To test for group effect, we implemented the one sample t-test on the extracted beta estimation.

Effect(s) tested

Nav-Math experiment: contrast were defined as: Navigation > Math; Math > Navigation; Navigation > Rest and Math > Rest.
Clock Navigation experiment: beta estimates of the GLM2 of the six fold effect and the other control fold effect.
If not differently specified, group effects were tested by performing one sample t-tests (within groups effects) or two sample t-tests (between groups effects) in both the experiments. Wilcoxon test was used for not normally distributed data.

Specify type of analysis: ☐ Whole brain ☐ ROI-based ☒ Both

Anatomical location(s) | Entorhinal cortex ROIs were cytoarchitectural defined (i.e., Brodmann's area 28, labeled as the ex vivo one) using Freesurfer (v7.1.1, https://surfer.nmr.mgh.harvard.edu/), and converted to the volumetric space using the 'mri_convert' function of the software.

Statistic type for inference
(See Eklund et al. 2016) | Nav-Math experiment: A voxel-level FDR corrected p = 0.05 was set as the threshold for multiple comparisons.

Correction | Nav-Math experiment: False Discovery Rate (FDR) correction; Clock Navigation experiment: family-wise error (FWE) correction

Models & analysis

n/a	Involved in the study
☒	☐ Functional and/or effective connectivity
☒	☐ Graph analysis
☒	☐ Multivariate modeling or predictive analysis
